# Changing the narrative: Socioeconomic determinants, not gun ownership, drive global homicide rates across 237 countries and territories

**DOI:** 10.1016/j.dialog.2025.100270

**Published:** 2025-12-23

**Authors:** Rae-Anne Kastle, Shelby Baxter, Christine Edomwande, An-Lin Cheng, Michael Moncure, Cuthbert Simpkins

**Affiliations:** a KCU College of Medicine, United States of America; b University of Kansas, United States of America; c UMKC School of Medicine, Departments of Surgery and Biomedical Sciences, United States of America; d University Health Department of Surgery, United States of America

**Keywords:** Intentional homicide, Gun ownership, Income inequality, Poverty, Socioeconomic determinants, Global health, Violence prevention, Maternal mortality

## Abstract

Intentional homicide is defined as the unlawful taking of a human life with the intent to cause death or serious injury. Gun ownership is often at the forefront of debates regarding IH, but few have explored this relationship worldwide. Our study seeks to fill this gap by examining the relationship between civilian gun ownership and IH rates globally, while also analyzing various socioeconomic determinants. To investigate this relationship, we conducted a retrospective, cross-national review using publicly available data reported from government websites and public health journals across 237 countries and territories. Spearman Correlation analysis, multivariable linear regression, and ANOVA were conducted with homicide rates as the dependent variable and were repeated after countries were stratified by gross national income. *P*-values <0·05 were considered significant. Civilian gun ownership did not correlate with IH rates with and without the stratification. The dependent variables that showed the strongest positive correlations with IH rates included Gini Index (r^2^ = 0·55; *p* < 0·0001) and maternal mortality ratio (r^2^ = 0·52; *p* < 0·0001). Linear regression showed that the Gini Index (r^2^ = 0·60; p < 0·0001) and poverty rate (r^2^ = 0·44; *p* = 0·033) can be used to calculate possible IH rates. Gini Index had a positive correlation with IH in lower-income countries. Globally, we found that the Gini Index and poverty rates were strongly associated with IH rates, while there was no correlation between civilian gun ownership. This suggests there are several underlying socioeconomic factors, rather than gun ownership alone, that contribute to IH globally.

## Highlights

• This study examined intentional homicide across 237 countries and territories.
• Unlike prior work, it included a wide range of socioeconomic and health indicators.
• Civilian gun ownership was not associated with global homicide rates.
• Income inequality and poverty had the strongest correlations to intentional homicide.
• Reducing inequality and improving maternal and child health may lower violence.

## 1 Introduction

Intentional homicide (IH) is defined as the unlawful taking of a human life with the intent to cause death or serious injury. IH includes killings due to interpersonal violence, domestic violence, and killings by armed groups, but not killings in armed conflict (e.g., war crimes/operations) [1]. Specifically, the United Nations Office on Drugs and Crime (UNODC) characterizes armed conflict as international conflicts between states or non-international conflicts between government and armed groups within a state. It is important to consider that definitions of intentional homicide and conflict-related deaths overlap. Three elements of IH, as defined by UNODC, are objective, subjective, and legal. The objective element is classified as the “killing of a person by another person,” and the subjective element includes the “intent of the perpetrator to kill or seriously injure” [2]. Lastly, the legal element considers the “perpetrator liable for the unlawful death” [2]. For instance, a fatal shooting of a home intruder in lawful defense would not be classified as intentional homicide, whereas a premeditated or retaliatory killing outside a combat zone would.

Homicide data is reported through different registries that collect information either from criminal justice systems or public health sources, generated by law enforcement or health authorities, respectively [2]. UNODC gathers homicide data from the former, and many countries also prefer this approach [2]. However, some countries, such as Mexico and Brazil, opt for public health data based on the International Classification of Diseases and Related Health Problems (ICD), often due to data quality issues or the unavailability of criminal justice data [2]. Because national criminal codes and reporting practices vary, definitional inconsistency remains a major source of measurement error across countries [3].

In 2021, there were an estimated 458,000 homicides that occurred throughout the world. Although this is likely an underestimation, it is equivalent to 52 deaths per hour [2]. When considering geographic variations, Africa accounted for the most considerable intentional homicide burden with 154,000 victims. The Americas followed at 109,000 victims, while Europe and Oceania reported 17,000 and 1000 deaths, respectively. In terms of homicide rates for each region, the Americas posed the greatest risk of being intentionally killed, with a rate of 15 per 100,000 [2].

Recent international data indicate that while global homicide rates have declined modestly over the past two decades, from approximately 6.9 per 100,000 people in 2000 to 5.8 in 2021; however, these reductions have occurred primarily in high-income regions. The Global Study on Homicide 2023 by the UNODC attributes persistent homicide burdens to socioeconomic inequality while noting that nations with greater income disparity continue to experience higher levels of interpersonal violence [4]. A similar study linked persistent socioeconomic disparity to elevated interpersonal and community violence, thus underscoring that inequality continues to mediate both the expression and persistence of violent behavior across populations on an international scale [5].

Gun ownership is often at the forefront of public health debates; however, there remains limited concrete evidence on how it may contribute to levels of intentional homicide globally. While several national studies have evaluated the association between civilian gun ownership and homicide, few have explored this relationship worldwide [6,7]. Additionally, other socioeconomic factors are overshadowed by gun violence. Efforts to reduce IH require an understanding of the social determinants of health, including healthcare quality, employment, housing, and food access, and their relationships to IH [8]. Understanding how these factors influence IH rates is crucial for developing effective legislation and prevention strategies.

Although national research has explored connections between violence, firearm availability, poverty, and income inequality, comprehensive cross-national studies are still rare. Our research aims to fill this gap by investigating the link between civilian gun ownership and IH rates worldwide. It also examines various socioeconomic factors and public health indicators to see how they might relate to IH levels globally. This study supports the World Health Organization's Global Plan of Action for Violence Prevention 2020–2030, particularly Objectives 1–4, which focus on enhancing data, increasing access to services, addressing social determinants, and fostering inclusive societies, and aligns with the United Nations Sustainable Development Goals 1 (No Poverty), 3 (Good Health and Well-Being), 10 (Reduced Inequalities), and 16 (Peace, Justice, and Strong Institutions).

## 2 Methods

This was a retrospective, cross-national review using publicly available international data to evaluate the relationship between various socioeconomic factors and intentional homicide rates across 237 countries/territories.

### 2.1 Search strategy and selection criteria

We examined data from various government sources, including datasets from the CIA World Factbook, the United Nations Office on Drugs and Crime (UNODC), the World Bank, Our World in Data (OWID), the World Health Organization (WHO), World Population Review, the United Nations Children's Fund (UNICEF), the Small Arms Survey, and the United Nations Development Programme (UNDP). We also examined data from global networks, including the International Coalition for Children with an Incarcerated Parent (INCCIP). Additionally, we utilized data from published public health articles, including The Happiness Report, Mental State of the World, and the Organized Crime Index. Since the data was obtained from datasets, informed consent was not required.

We included all countries regardless of the number of socioeconomic variables available. Sources were included if they reported comparable data across a broad set of countries and territories, and excluded if fewer than 50 countries or territories were represented for a given variable. Data from the datasets were organized into an Excel spreadsheet with socioeconomic factors as columns and countries as rows. If a dataset lacked any value for a specific country, the corresponding cell was left blank. The most recent value reported in the dataset was recorded, spanning from 1970 to 2024. Lastly, unions of countries were included alongside individual countries in the list, and the data were reported accordingly. For example, datasets reporting values for the United Kingdom were recorded in that row; however, data for Scotland, Northern Ireland, England, and Wales were recorded in their respective rows, if reported separately. This also applied to the Channel Islands (Guernsey and Jersey) and the Palestinian territories (Gaza and the West Bank).

We focused on comparing the homicide rate (per 100,000) [[9], [10], [11], [12], [13]], the dependent variable, to a wide range of social and economic indicators, the independent variables. The social factors that were assessed included infant mortality rate (deaths/ per 1000 live births) [14,15], life evaluations of happiness [16], the Mental Health Quotient [17], civilian gun ownership (firearms per 100 capita) [18], birth rates (births per 1000 population) [19,20], maternal mortality ratio (deaths per 100,000 live births) [21], life expectancy at birth (years) [22,23], suicide rates (per 100,000 population) [24], The Human Development Index (HDI) [25], child labor (%) [26], and children in detention (per 100,000) [27].

The economic factors included the Gini index (%) [28], unemployment rates (%) [29], GDP (USD per capita) [30], criminality average [31], resilience average [31], inflation rates (%) [32], education expenditures (% of GDP) [33], poverty rate (%) [34], the poverty headcount ratio at $2·15 a day (percentage of the population living under the extreme poverty line ($2·15/day)) [35], the Multidimensional Poverty Index (MPI) [36], and the crime rate (per 100,000) [37]. The Gini Index measures the extent to which a country's income or wealth distribution deviates from perfect equality among its residents. The Gini Index ranges from 0 to 1, with values close to 1 indicating greater income inequality [38].

### 2.2 Data analysis

Data analysis was performed using XLSTAT in Microsoft Excel. First, a Spearman Correlation analysis was conducted with pairwise deletion to remove singular missing data points of the independent variables and intentional homicide rates. *P*-values <0·05 were considered significant. Next, countries with more than half of the variables missing and with insignificant variables, as determined by the Spearman Correlation, were excluded from the analysis, except for civilian gun ownership (firearms per 100 capita). This is due to exploring the common narrative that more guns lead to more crime, and to assess possible interactions with other socioeconomic variables.

A multivariable linear regression was performed with homicide rates as the outcome variable. Multicollinearity was assessed using the Variance Inflation Factor (VIF). Finally, ANOVA was conducted on the remaining independent variables to investigate the relationship between socioeconomic factors and intentional homicide rates. Under the missing data tab, “remove observations for each Y separately” was selected to retain as many data points as possible. *P*-values <0·05 were also used as the cutoff for significance in the second analysis. The number of data points per independent variable, P-values, and correlation coefficients were documented in a separate table (Table 1).

**Table 1:** Spearman correlations and multivariable linear regression of socioeconomic indicators associated with intentional homicide rates across 237 countries/territories.

	Spearman Correlation			Linear Regression		
	Observations	P-Value < 0·05	Correlation (r^2^)	Observations	Type III SS P-Value < 0·05	Correlation (r^2^)
Gini Index (%)	179	< 0·0001	0·554	111	< 0·0001	0·60
Unemployment Rate (%)	209	0·001	0·231	111	0·35	0·25
GDP (USD per Capita)	221	< 0·0001	−0·394	111	0·67	−0·31
Criminality Average	193	0·006	0·196	111	0·31	0·31
Resilience Average	193	< 0·0001	−0·339	111	0·36	−0·19
Inflation Rate (%)	219	0·095	0·114	–	–	–
Education expenditure (% GDP)	196	0·124	−0·111	–	–	–
Poverty Rate (%)	157	< 0·0001	0·470	111	0·033	0·44
Poverty headcount ratio at $2·15 a day (2017 PPP) (% of population)	168	< 0·0001	0·464	111	0·11	0·24
Multidimensional Poverty Index	110	0·036	0·200	–	–	–
Crime Rate (per 100,000)	141	< 0·0001	0·636	111	–	–
Household Crowding (Millions)	194	0·088	0·123	–	–	–
Infant Mortality Rate (deaths/1000 births)	227	< 0·0001	0·383	111	0·083	0·18
Life Evaluations of Happiness	143	< 0·0001	−0·385	111	0·085	−0·14
Mental Health Quotient	57	0·031	0·287	–	–	–
Civilian Guns (per 100 Capita)	225	0·355	−0·062	111	0·97	−0·073
Birth Rate (births/1000 population)	228	< 0·0001	0·308	111	0·51	0·20
Maternal Mortality (deaths/100,000 live births)	186	< 0·0001	0·516	111	0·30	0·21
Life Expectancy at Birth (years)	228	< 0·0001	−0·362	111	0·41	−0·27
Suicide Rate (per 100,000 population)	184	0·369	−0·067	–	–	–
Human Development Index	193	< 0·0001	−0·424	111	0·11	−0·29
Child Labor	99	0·822	−0·023	–	–	–
Children in Detention (100,000)	160	0·035	0·167	111	0·79	0·10

Next, the countries were stratified using the World Bank Country and Lending Groups income classification. Countries are classified by Gross National Income (GNI) per capita in USD for the 2026 fiscal year and are divided into four income groups [39]. These groups include low-income ($1135 or less), lower-middle-income ($1136 to $4495), upper-middle-income ($4496 to $13,935), and high-income ($13,935 or more) economies [39]. After stratification, data analysis, including Spearman Correlation and linear regression, was repeated. *P*-values <0·05 were considered significant.

### 2.3 Role of the funding source

Funding was provided by the University of Missouri-Kansas City School of Medicine, Sosland-Missouri Endowment for Trauma Services. Funding for this study was used for data analysis but did not affect the study design, data collection, data interpretation, report writing, or the decision to submit the manuscript for publication.

## 3 Results

### 3.1 Spearman correlation

Surprisingly, we found that civilian gun ownership (firearms per 100 capita) (r^2^ = −0·062; *p* = 0·36), as shown in Table 1, did not correlate with the homicide rate. Other variables that were not statistically significant included the inflation rate (%), education expenditure (% of GDP), the suicide rate (per 100,000 population), and child labor rates (%). The variable with the strongest positive correlation to the intentional homicide rate was the Gini Index (r^2^ = 0·55; *p* < 0·0001), closely followed by the maternal mortality ratio (deaths per 100,000 live births) (r^2^ = 0·52; p < 0·0001). The variables with weak positive correlations included the poverty rate, poverty headcount ratio at $2·15 a day, infant mortality rate, birth rate, mental health quotient, percentage of children with an imprisoned parent, unemployment rate, the Multidimensional Poverty Index, criminality average, and children in detention (per 100,000). The variable with the strongest negative correlation to the intentional homicide rate was the Human Development Index (r^2^ = −0·42; *p* < 0·0001). The weaker negative correlations that followed included GDP per capita, Life Evaluations of Happiness, life expectancy at birth, and the resilience average (Table 1).

### 3.2 Linear regression

**Fig. 1:**
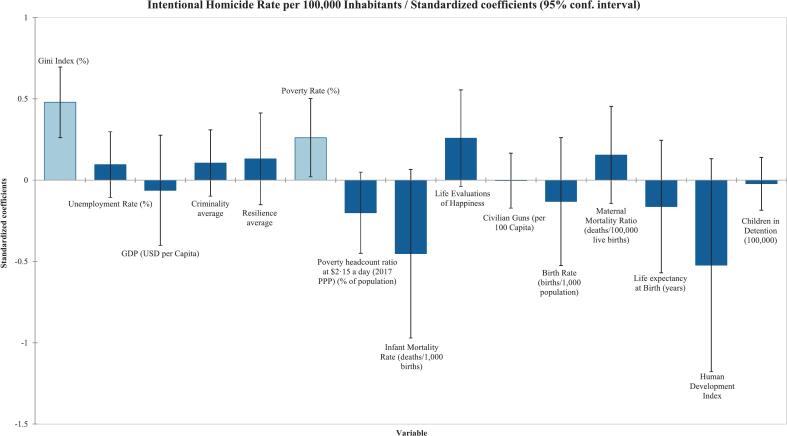
Standardized coefficients from multivariable linear regression of socioeconomic factors associated with intentional homicide rates. Each bar represents standardized regression coefficients, with error bars showing 95 % confidence intervals.

According to the Type III Sum of Squares analysis, it was found that the Gini Index and poverty rate were statistically significant, with correlation coefficients of r^2^ = 0·60 (*p* < 0·0001) and r^2^ = 0·44 (*p* = 0·033), respectively, as shown in Table 1. The ANOVA also had a p < 0·0001. Fig. 1 displays the regression coefficients with 95 % confidence interval error bars, indicating a strong positive correlation between the Gini index and poverty rate with homicide. The unemployment rate (%), GDP (USD per capita), criminality average, resilience average, poverty headcount ratio at $2·15 a day (% of population), infant mortality rate (deaths per 1000 live births), Life Evaluations of Happiness, birth rate (births per 1000 population), maternal mortality ratio (deaths per 100,000 live births), life expectancy at birth (years), Human Development Index, children in detention (per 100,000), and civilian gun ownership (firearms per 100 capita) were not found to be statistically significant in the linear regression as reported in Table 1 and shown in Fig. 1.

### 3.3 Stratified spearman correlation

Following stratification, 25 countries have low-income economies, 50 have low- and middle-income economies, 54 have upper-middle-income economies, and 87 have high-income economies. The respective countries and their stratification can be found in the appendix.

Among low-income countries, the Gini index (%) showed a positive correlation (r^2^ = 0·525) with IH (*p* = 0·013). No other variables were found to be statistically significant for low-income countries, as shown in Table 2. The low-middle-income economy countries had the strongest positive correlation between crime rate (per 100,000) and IH (r^2^ = 0·548) (*p* = 0·002). The weak positive correlations occurred between Gini index (%), poverty headcount ratio of $2·15 a day (% of population), Mental Health Quotient, poverty rate (%), maternal mortality ratio (deaths/100,000 live births), and infant mortality rate (deaths/1000 births) with IH (Table 2). Negative correlations to IH rates were discovered with life expectancy at birth (years) (r^2^ = −0·495) (*p* = 0·0004) and the Human Development Index (r^2^ = −0·336) (p = 0·018).

**Table 2:** Spearman correlation of socioeconomic indicators associated with intentional homicide rates of 216 countries and territories stratified by World Bank income classifications.ǂ

	Low-Income^A^			Low-Middle Income^B^			Middle-High Income^C^			High-Income^D^		
	Obs.	P-Value < 0·05	Correlation (r^2^)	Obs.	P-Value < 0·05	Correlation (r^2^)	Obs.	P-Value < 0·05	Correlation (r^2^)	Obs.	P-Value < 0·05	Correlation (r^2^)
Gini Index (%)	22	0·013	0·525	49	0·001	0·481	47	< 0·0001	0·624	56	0·008	0·353
Unemployment Rate (%)	25	0·874	−0·033	47	0·177	0·200	51	0·429	0·113	77	0·0003	0·405
GDP (USD per Capita)	25	0·303	−0·214	49	0·322	−0·144	54	0·247	−0·160	84	< 0·0001	−0·461
Criminality average	25	0·587	0·114	49	0·662	0·064	53	0·243	0·163	64	0·839	−0·026
Resilience average	25	0·430	−0·164	49	0·069	−0·262	53	0·819	0·032	64	0·976	−0·004
Inflation rate (%)	24	0·639	−0·100	49	0·733	0·050	54	0·897	0·018	81	0·546	0·068
Education Expenditure (% GDP)	21	0·118	−0·352	48	0·731	−0·051	46	0·109	0·239	74	0·771	−0·034
Poverty Rate (%)	22	0·372	0·199	49	0·003	0·418	42	0·017	0·369	42	0·069	0·284
Poverty headcount ratio at $2·15 a day (2017 PPP) (% of population)	21	0·364	0·208	48	0·001	0·472	49	0·007	0·381	48	0·018	0·342
Multidimensional Poverty Index	18	0·441	0·193	44	0·091	0·258	41	0·0003	0·539	5	0·350	−0·600
Crime Rate (per 100,000)	9	0·250	−0·433	31	0·002	0·548	39	0·0002	0·562	59	< 0·0001	0·555
Household Crowding (Millions)	24	0·779	0·060	49	0·619	−0·073	52	0·763	−0·043	65	0·389	−0·108
Infant Mortality Rate (deaths/1000 births)	24	0·844	0·043	50	0·004	0·401	54	0·591	0·075	87	< 0·0001	0·420
Life Evaluations of Happiness	16	0·991	−0·004	39	0·871	−0·027	37	0·671	0·072	50	0·218	0·177
Mental Health Quotient	3	1·000	0·500	19	0·047	0·463	14	0·146	−0·411	20	0·047	0·451
Civilian Guns (per 100 Capita)	25	0·993	0·002	50	0·164	0·200	54	0·589	0·075	86	0·893	−0·015
Birth Rate (births/1000 population)	25	0·542	0·128	49	0·202	0·185	54	0·164	0·192	87	0·022	0·245
Maternal Mortality Ratio (deaths/100,000 live births)	25	0·234	0·246	49	0·004	0·409	50	0·001	0·445	58	0·0003	0·459
Life expectancy at Birth (years)	25	0·785	−0·057	49	0·0004	−0·495	54	0·618	−0·069	87	0·001	−0·338
Suicide rates (per 100,000 population)	24	0·314	0·214	50	0·172	0·196	50	0·963	0·007	58	0·513	0·087
Human Development Index	24	0·470	−0·154	50	0·018	−0·336	53	0·057	−0·263	64	0·005	−0·349
Child Labor (%)	19	0·965	−0·011	39	0·105	0·263	31	0·969	0·007	8	0·536	0·262
Children in Detention (100,000)	16	0·652	0·122	35	0·565	−0·100	45	0·022	0·341	60	0·509	0·087

ǂ Check Appendix for country classifications.

IH rates in the upper-middle economy countries had the strongest positive correlation with Gini index (%) (r^2^ = 0·624) (*p* < 0·0001), crime rate (per 100,000) (r^2^ = 0·562) (p = 0·0002), and Multidimensional Poverty Index (r^2^ = 0·539) (p = 0·0003). Other weaker positive correlations to IH included maternal mortality ratio (deaths/100,000 live births), poverty headcount ratio at $2·15 a day (% of population), poverty rate (%), and children in detention (per 100,000), shown in Table 2. There were no negative correlations for this stratification. Lastly, the high-income economy countries showed that crime rate (per 100,000) had the strongest positive relationship with IH rates (r^2^ = 0·555) (*p* < 0·0001). The variables with a weaker positive correlation, which can be found in Table 2, include maternal mortality ratio (deaths/100,000 live births), Mental Health Quotient, infant mortality rate (deaths/1000 births), unemployment rate (%), Gini index (%), poverty headcount ratio at $2·15 a day (% of population), and birth rate (births/1000 population). Among the negative correlations to IH, GDP (USD per capita) was the strongest with p < 0·0001 and r^2^ = −0·461. The weaker negative correlations included life expectancy at birth (years) and the Human Development Index (Table 2).

### 3.4 Stratified linear regression

**Fig. 2:**
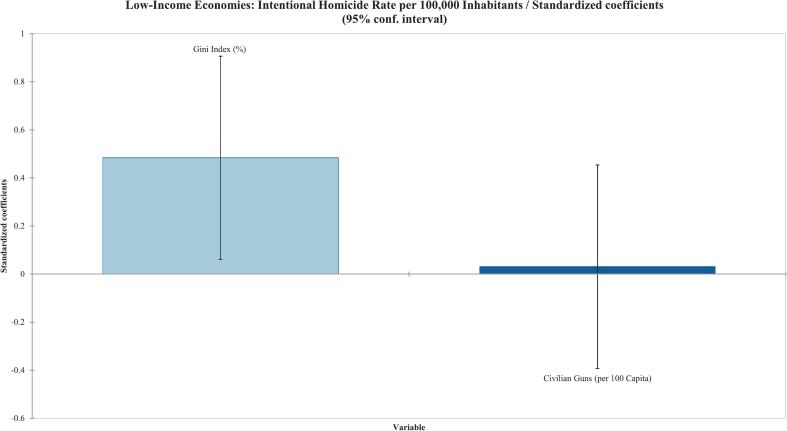
Standardized coefficients from multivariable linear regression of socioeconomic factors associated with intentional homicide rates in stratified low-income countries and territories. Each bar represents standardized regression coefficients, with error bars showing 95 % confidence intervals.

**Fig. 3:**
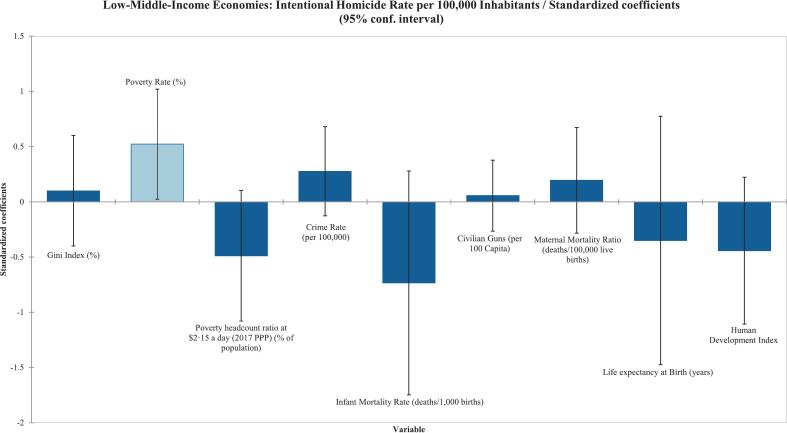
Standardized coefficients from multivariable linear regression of socioeconomic factors associated with intentional homicide rates in stratified low-middle-income countries and territories. Each bar represents standardized regression coefficients, with error bars showing 95 % confidence intervals.

**Fig. 4:**
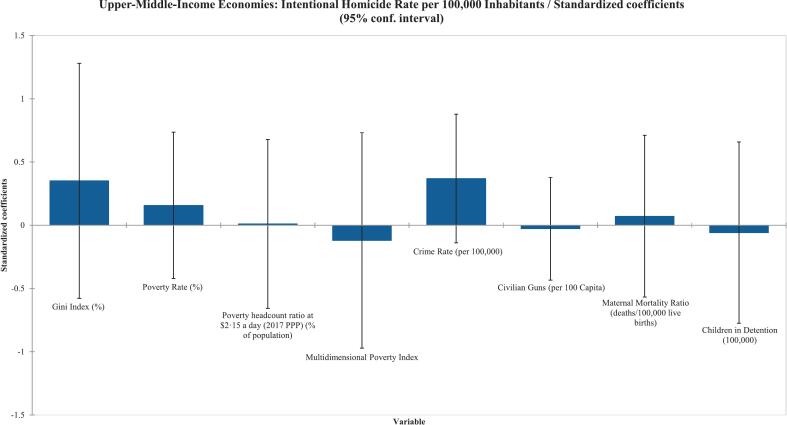
Standardized coefficients from multivariable linear regression of socioeconomic factors associated with intentional homicide rates in stratified upper-middle-income countries and territories. Each bar represents standardized regression coefficients, with error bars showing 95 % confidence intervals.

**Fig. 5:**
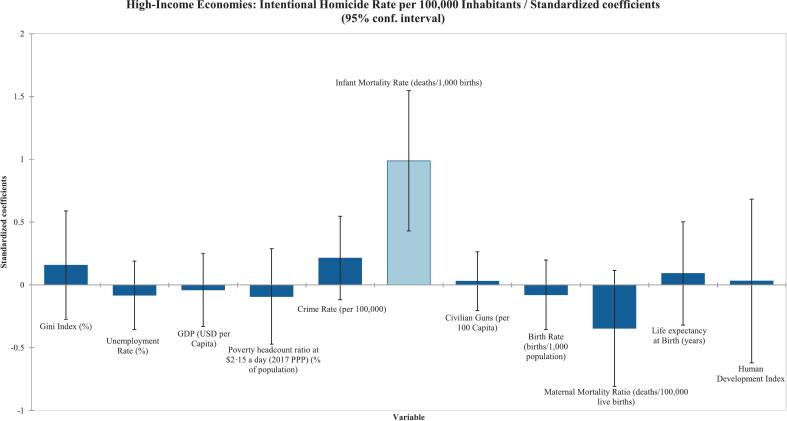
Standardized coefficients from multivariable linear regression of socioeconomic factors associated with intentional homicide rates in stratified high-income countries and territories. Each bar represents standardized regression coefficients, with error bars showing 95 % confidence intervals.

Among low-income countries, the Gini index (%) was the only variable found to be statistically significant in the Type III Sum of Squares analysis, with a correlation coefficient of 0·481 (*p* = 0·027), as shown in Table 3 and reflected in Fig. 2. Similarly, poverty rate (%) was the only variable found to be statistically significant for the low-middle-income economy countries (r^2^ = 0·657) (p = 0·027) as shown in Table 3 and Fig. 3. Surprisingly, the upper-middle-income countries had no variables that were statistically significant in the Type III sum-of-squares analysis (Table 3) (Fig. 4). Finally, the high-income countries showed statistical significance in infant mortality rate (deaths/1000 births), as shown in Table 3 and Fig. 5. Civilian gun ownership (firearms per 100 capita) was not significant in any analysis.

**Table 3:** Linear regression of socioeconomic indicators associated with intentional homicide rates across 237 countries/territories stratified by World Bank income classifications.ǂ

		Observations	Type III SS P-Value < 0·05	Correlation (r^2^)
Low-Income^A^	Gini Index (%)	22	0·027	0·481
	Civilian Guns (per 100 Capita)	22	0·880	−0·018
Low-Middle-Income^B^	Gini Index (%)	29	0·679	0·307
	Poverty Rate (%)	29	0·041	0·657
	Poverty headcount ratio at $2·15 a day (2017 PPP) (% of population)	29	0·099	0·245
	Crime Rate (per 100,000)	29	0·167	0·620
	Infant Mortality Rate (deaths/1000 births)	29	0·146	0·199
	Civilian Guns (per 100 Capita)	29	0·718	0·010
	Maternal Mortality Ratio (deaths/100,000 live births)	29	0·404	0·365
	Life expectancy at Birth (years)	29	0·523	−0·360
	Human Development Index	29	0·180	−0·389
Upper-Middle-Income^C^	Gini Index (%)	26	0·436	0·662
	Poverty Rate (%)	26	0·573	0·488
	Poverty headcount ratio at $2·15 a day (2017 PPP) (% of population)	26	0·973	0·467
	Multidimensional Poverty Index	26	0·770	0·243
	Crime Rate (per 100,000)	26	0·144	0·666
	Civilian Guns (per 100 Capita)	26	0·886	−0·161
	Maternal Mortality Ratio (deaths/100,000 live births)	26	0·815	0·426
	Children in Detention (100,000)	26	0·867	0·181
High-Income^D^	Gini Index (%)	45	0·463	0·611
	Unemployment Rate (%)	45	0·541	0·209
	GDP (USD per Capita)	45	0·787	-0·325
	Poverty headcount ratio at $2·15 a day (2017 PPP) (% of population)	45	0·628	0·384
	Crime Rate (per 100,000)	45	0·199	0·616
	Infant Mortality Rate (deaths/1000 births)	45	0·001	0·727
	Civilian Guns (per 100 Capita)	45	0·797	-0·016
	Birth Rate (births/1000 population)	45	0·570	0·303
	Maternal Mortality Ratio (deaths/100,000 live births)	45	0·137	0·471
	Life expectancy at Birth (years)	45	0·652	-0·397
	Human Development Index	45	0·922	-0·602

ǂ Check Appendix for country classifications.

## 4 Discussion

In a global analysis of 237 countries and territories, we found that the Gini Index and poverty rates were strongly associated with IH rates. Gini Index was strongly associated with IH rates in lower-income countries even after stratification by GNI. However, there was no correlation between civilian gun ownership and IH rates in all analyses, which is contrary to many national-level public health debates. We also identified several variables that positively correlated with IH, including maternal mortality ratio, poverty headcount ratio at $2·15 a day, infant mortality rate, birth rate, Mental Health Quotient, Life Evaluations of Happiness, unemployment rate, Multidimensional Poverty Index, crime rate, criminality average, and children in detention.

Current literature rarely discusses the relationship between civilian gun ownership and IH globally, but several studies in the United States highlight the complex correlation between the two. For example, a study published in 2013 by Siegel et al. examined gun ownership by proxy measurement and firearm homicide rates from 1981 to 2010 [8]. They found gun ownership to be a significant predictor of firearm homicide rates, with a 1 % increase in gun ownership corresponding to a 0.9 % increase in homicide rates [8]. On a global scale, a study in 2000 investigated firearm availability and homicide rates among 26 high-income countries [7]. They used two proxies for gun availability: the percentage of suicides involving a firearm and the Cook Index, which measures the percentage of suicides and homicides involving firearms. Their analysis revealed a positive correlation between total homicide rates and the two proxies for gun availability, even when the United States was excluded.

Our study differs from most in two key ways. First, we did not rely on a proxy to estimate gun ownership; instead, we collected data directly from the Small Arms Survey on civilian gun ownership, which enhanced the significance of our cross-national study. The Small Arms Survey compiles national estimates of civilian firearm possession through a mixed-methods approach that incorporates household surveys, administrative records, expert assessment, and inference from comparable nations. Our analysis used the consolidated national estimates, which include both survey-based and expert-derived figures. Second, we examined several other social and economic variables that might influence IH rates globally. Our research employs a multifaceted approach, considering variables such as the Gini Index and poverty levels, rather than focusing solely on civilian gun ownership.

It is important to consider one's motive to own a gun, such as for personal protection, hunting, or sport, which may vary by country. Specifically in the United States, one study concluded that the primary reason Americans own guns is for personal protection, especially at-home protection. Out-of-home protection is becoming increasingly common as well, especially in states where open-carry laws are permitted [40]. Gun culture in Switzerland widely differs from that in the United States, as Swiss gun owners predominantly use them for hunting, sport, and defending their country [41]. Globally, research is scarce on understanding why people in certain countries own guns, but expanding on this could offer valuable insights into the relationship between civilian gun ownership and IH.

The term Gini Index was first introduced in 1912 by Corrado Gini and further studied by M.O. Lorenz [42]. Specifically, Lorenz developed the Lorenz Curve, a tool for visualizing wealth distribution within a population. The Gini Index ranges from 0 to 1, where 0 indicates low income inequality, and 1 indicates high income inequality [42]. Gini Indices differ across countries, and various socioeconomic factors such as IH rates, maternal mortality, and poverty levels may be influenced by a country's index. There is a significant correlation between the Gini Index and IH levels worldwide, suggesting that higher income inequality may influence increased homicide rates.

While the link between income inequality and IH rates is well established, the underlying explanation remains debated due to numerous confounding factors [1]. Seemingly, in countries with high Gini indices, a small elite continues to control the majority of wealth, while others face hardships, such as difficulty paying bills or living in food deserts. Two established hypotheses have been proposed to explain the correlation between income inequality and IH: the psychosocial and the neomaterialist hypotheses [43]. The psychosocial hypothesis focuses on social capital, such as trust within a community, whereas the neomaterialist hypothesis focuses on the effects of income inequality on political and economic policies [43]. Together, these hypotheses illustrate that if a society has less trust and investment in public services and infrastructure, it can contribute to an increase in violent behaviors, such as IH [43].

Additionally, poverty rates are also strongly linked to IH in our analysis. This connection has been shown in the literature, with a 30-year Brazilian cohort study finding that lifelong poverty is associated with homicide rates, particularly in early adulthood [44]. Once again, theories explaining the relationship between these two are speculative. However, several hypotheses exist, such as a lack of employment opportunities, segregated low-income neighborhoods, and gang violence control [44]. The relationship between poverty and violence often results from a lack of support and resources for youth. Overall, it is reasonable to suggest that limited access to basic needs may lead to criminal behavior, which can escalate into violence [45].

Maternal mortality is a sensitive indicator of a society's development and healthcare infrastructure [46]. Our study found a positive correlation between maternal mortality ratios and IH rates globally, indicating that countries with higher maternal mortality ratios tend to have more IH. Increased maternal mortality in developing countries can often be linked to a lack of maternal education, lower socioeconomic status, and limited access to maternal care [46]. A common theme in our findings suggests that a country's lack of resources and overall health is consistent with more violent behaviors, which may explain the higher IH rates.

Lastly, the Human Development Index (HDI) is a measure created by the United Nations Development Programme to assess a country's overall development, not just its economic growth [25]. It includes three dimensions: a long and healthy life, knowledge, and a decent standard of living [25]. Our analysis found that HDI was negatively correlated with IH rates, which was expected based on previous research [47]. Therefore, the less educated, healthy, and economically developed a country is, the more likely it is to experience increases in IH. This supports the recurring idea that socioeconomic disadvantage raises the risk of violence [48].

These findings align with ecological and social disorganization theories, which postulate that structural disadvantage and inequality weaken social cohesion and foster violence. Classic work by Shaw and McKay (1942) and Sampson et al. (1997) demonstrates how neighborhood-level deprivation erodes collective efficacy [49,50]. Similarly, cross-national research has linked income inequality to homicide and interpersonal violence [43]. The World Health Organization's ecological model further supports this multilevel perspective, emphasizing how individual, community, and societal factors interact to shape violence risk [51].

### 4.1 Limitations

Taken together, these findings highlight the complex socioeconomic foundations of global homicide patterns. Since this study is retrospective in nature, it limits the ability to establish causation and prevents assessment of long-term outcomes. Although associations between socioeconomic variables were identified, causal relationships cannot be confirmed. Despite efforts to compile complete datasets, many variables had missing values, which may introduce bias or reduce statistical power even when using pairwise deletion. Additionally, data were sourced from multiple international sources that vary in collection standards, time frames, and methods, potentially affecting cross-national comparability. The linear regression analysis includes only countries with complete data for each variable, significantly reducing the number of observations per stratification. This smaller sample size could introduce selection bias, underscoring the need for standardized, high-quality international datasets to improve future cross-national analyses.

The Small Arms Survey data lacks disaggregation (such as distinguishing between legal and illegal ownership), which could explain why the correlation between gun ownership and intentional homicide appears nonsignificant. Consequently, reported ownership values should be viewed as estimates that may be affected by under- or over-reporting, differences in data-collection years, and variations across national registration systems. These methodological differences present an inherent challenge when comparing firearm prevalence across countries. It's also important to recognize that defining IH is not always straightforward, as some countries may or may not report deaths from armed conflicts as IH. The applicability of these findings should be approached cautiously, especially for underserved or conflict-affected populations where socioeconomic data is limited or unreliable. Lastly, although multicollinearity was addressed, unmeasured confounding variables might have influenced the results.

### 4.2 Future directions

Future research should expand on these findings by employing prospective or longitudinal study designs to examine the relationships among socioeconomic factors, health conditions, and changes in homicide rates over time. Greater integration of standardized international datasets would improve comparability and reduce bias across regions. Unmeasured contextual factors such as gang violence, religious ecologies, and cultural norms may influence violence patterns. Further studies could explore moral and cultural contexts to understand their influence on violence, including factors like gang violence and religion. Additionally, targeted investigations in underserved or conflict-affected populations are necessary to determine if these findings are applicable broadly. Ultimately, future research should assess the impact of policy interventions designed to reduce income inequality, alleviate poverty, and enhance maternal and child health on measurable declines in violence. By incorporating both structural and sociocultural factors, future research can help develop comprehensive, evidence-based strategies to prevent global homicide.

## CRediT authorship contribution statement

**Rae-Anne Kastle:** Writing – review & editing, Writing – original draft, Validation, Software, Methodology, Investigation, Formal analysis, Data curation, Conceptualization. **Shelby Baxter:** Writing – review & editing, Writing – original draft, Validation, Resources, Investigation, Conceptualization. **Christine Edomwande:** Writing – review & editing, Writing – original draft, Visualization. **An-Lin Cheng:** Writing – review & editing, Validation, Software, Formal analysis. **Michael Moncure:** Writing – review & editing, Supervision, Project administration, Investigation. **Cuthbert Simpkins:** Writing – review & editing, Writing – original draft, Supervision, Investigation, Funding acquisition, Data curation, Conceptualization.

## Declaration of competing interest

We declare no competing interests.

## Glossary

Birth rate: The number of live births per 1000 population in a given year, used as a measure of population growth.
Child labor: The engagement of children in work that deprives them of their childhood, education, or is harmful to their physical or mental development, typically measured as the percentage of children aged 5–17 involved in labor.
Civilian gun ownership: The number of firearms held by civilians, measured per 100 persons, including both registered and unregistered weapons.
Criminality average: A composite score developed by the Global Organized Crime Index representing the overall level of criminal activity, including violence, trafficking, and organized crime within a country.
Education expenditures: The percentage of a nation's gross domestic product (GDP) spent on education at all levels, used as an indicator of government investment in human capital.
Gini Index: A measure of income inequality within a population, ranging from 0 (perfect equality) to 1 (perfect inequality).
Gross Domestic Product (GDP): The total monetary value of all goods and services produced within a country's borders during a specific period, often used as a measure of economic performance.
Gross National Income (GNI): The total income earned by a country's residents and businesses and is used to measure a country's economic health.
Human Development Index (HDI): A composite measure of national development combining indicators of life expectancy, education, and income per capita.
Infant mortality rate (IMR): The number of infant deaths (under one year of age) per 1000 live births in a given year.
Inflation rate: The annual percentage increase in the average price level of goods and services in an economy, reflecting changes in purchasing power.
Intentional homicide (IH): The unlawful death of a person purposefully inflicted by another individual, excluding deaths from armed conflict or negligent acts.
Interpersonal violence (IPV): The use of physical force or power against another person that results in injury, death, or psychological harm, often occurring between family members, partners, or acquaintances.
Maternal mortality ratio (MMR): The number of maternal deaths per 100,000 live births during a specified time period.
Multidimensional Poverty Index (MPI): A composite indicator assessing deprivations in health, education, and living standards at the household level.
Multivariable linear regression: A statistical method that models the relationship between one dependent variable and multiple independent variables to identify predictors while controlling for potential confounders.
Poverty rate: The percentage of a population living below an established poverty threshold, such as the international poverty line defined by the World Bank.
Resilience average: A composite score from the Global Organized Crime Index representing a country's ability to resist, adapt to, and recover from organized crime and social disruption.
Small Arms Survey: A research project based in Geneva that collects and analyzes global data on firearm ownership, armed violence, and small arms trafficking.
Socioeconomic factors: Conditions that influence individual and group differences in health and well-being, including income, education, employment, social class, and access to resources.

## Appendix A Country stratification

^A^ Countries include Afghanistan, Burkina Faso, Burundi, Central African Republic, Chad, Congo (Democratic Republic of the), Eritrea, Gambia, Guinea-Bissau, Korea (DPR), Liberia, Madagascar, Malawi, Mali, Mozambique, Niger, Russia, Sierra Leone, Somalia, South Sudan, Sudan, Syria (Syrian Arab Republic), Togo, Uganda, and Yemen.

^B^ Countries include Angola, Bangladesh, Benin, Bhutan, Bolivia, Cambodia, Cameroon, Comoros, Congo (Republic of the), Ivory Coast (Cote d'Ivoire), Djibouti, Egypt, Eswatini, Ghana, Guinea, Haiti, Honduras, India, Jordan, Kenya, Kiribati, Kyrgyzstan, Laos, Lebanon, Lesotho, Mauritania, Micronesia, Morocco, Myanmar (Burma), Namibia, Nepal, Nicaragua, Nigeria, Palestine (Gaza Strip and West Bank), Pakistan, Papua New Guinea, Philippines, São Tomé and Príncipe, Senegal, Solomon Islands, Sri Lanka, Tajikistan, Tanzania (United Republic of), East Timor (Timor-Leste), Tunisia, Uzbekistan, Vanuatu, Viet Nam, Zambia, and Zimbabwe.

^C^ Countries Include Albania, Algeria, Argentina, Armenia, Azerbaijan, Belarus, Belize, Bosnia and Herzegovina, Botswana, Brazil, Cape Verde (Cabo Verde), China, Colombia, Cuba, Dominica, Dominican Republic, Ecuador, El Salvador, Equatorial Guinea, Fiji, Gabon, Georgia, Grenada, Guatemala, Indonesia, Iran, Iraq, Jamaica, Kazakhstan, Kosovo, Libya, Malaysia, Maldives, Marshall Islands, Mauritius, Mexico, Moldova (Republic of), Mongolia, Montenegro, North Macedonia, Paraguay, Peru, Samoa, Serbia, South Africa, Saint Lucia, Saint Vincent and the Grenadines, Suriname, Thailand, Tonga, Turkey, Turkmenistan, Tuvalu, and Ukraine.

^D^ Countries Include American Samoa, Andorra, Antigua and Barbuda, Aruba, Australia, Austria, Bahamas, Bahrain, Barbados, Belgium, Bermuda, British Virgin Islands, Brunei, Bulgaria, Canada, Caymen Islands, Channel Islands (Guernsey & Jersey), Chile, Costa Rica, Croatia, Curaçao, Cyprus, Czech Republic (Czechia), Denmark, Estonia, Faroe Islands, Finland, France, French Polynesia, Germany, Gibraltar, Greece, Greenland, Guam, Guyana, Hong Kong, Hungary, Iceland, Ireland, Isle of Man, Israel, Italy, Japan, Korea (Republic of Korea), Kuwait, Latvia, Liechtenstein, Lithuania, Luxembourg, Macau, Malta, Monaco, Nauru, Netherlands, New Caledonia, New Zealand, Northern Mariana Islands, Norway, Oman, Palau, Panama, Poland, Portugal, Puerto Rico, Qatar, Romania, Russia, San Marino, Saudi Arabia, Seychelles, Singapore, Saint Martin, Slovakia, Slovenia, Spain, Saint Kitts and Nevis, Saint Martin, Sweden, Switzerland, Taiwan, Trinidad and Tobago, Turks and Caicos Islands, United Arab Emirates, United Kingdom (England, Wales, Scotland, & Northern Ireland), United States, Uruguay, and U.S. Virgin Islands.

### Data sharing

All data sets used in the study will be made available to others upon publication and are cited in the references at the end of this manuscript.
